# Considering the Genetic Architecture of Hypoplastic Left Heart Syndrome

**DOI:** 10.3390/jcdd9100315

**Published:** 2022-09-21

**Authors:** John W. Belmont

**Affiliations:** Departments of Molecular and Human Genetics and Pediatrics, Baylor College of Medicine, Genetics & Genomics Services, Inc., Houston, TX 77096, USA; jbelmont@bcm.edu

**Keywords:** hypoplastic left heart syndrome, cardiovascular malformations, birth defects, genetics, genomics, inborn errors of development

## Abstract

Hypoplastic left heart syndrome (HLHS) is among the most severe cardiovascular malformations and understanding its causes is crucial to making progress in prevention and treatment. Genetic analysis is a broadly useful tool for dissecting complex causal mechanisms and it is playing a significant role in HLHS research. However, unlike classical Mendelian disorders where a relatively small number of genes are largely determinative of the occurrence and severity of the disease, the picture in HLHS is complex. De novo single-gene and copy number variant (CNV) disorders make an important contribution, but there is emerging evidence for causal contributions from lower penetrance and common variation. Integrating this emerging knowledge into clinical diagnostics and translating the findings into effective prevention and treatment remain challenges for the future.

## 1. Evidence for Important Genetic Components in HLHS

### 1.1. Background and Motivation

It is a truism that progress in preventing and treating diseases depends on understanding their causes. Palliative surgical and medical treatment for HLHS is long and arduous [1], and mortality much higher than other congenital heart diseases (CHD) [2]. HLHS is also among the most costly CHD to manage [3,4], creating long-term economic burdens for families and the entire health system. The underlying etiologies of hypoplastic left heart syndrome (HLHS) deserve careful scrutiny not only for these reasons but also for their broader impact. HLHS can be viewed as a classic inborn error of development [5] and so it is also representative of many other severe birth defects. HLHS etiologies include both genetic and non-genetic factors [6]. Presumably the non-genetic factors disturb cardiac development by acting on the same cells and developing structures as the genetic abnormalities. This reminds us that the resulting anatomic defects are caused by disturbed processes not genes [7]. We should expect combinations of factors rather than single simple mechanisms. Dissecting the cell and developmental processes that result in HLHS using genetic and multi-omic technologies will be broadly informative as a guide to solving other similar disease problems.

### 1.2. Familiality

HLHS and congenital left-sided lesions are marked by family clustering with an estimated relative recurrence rate of 12.9-fold [8]. Recurrences in sibships are well-known [9]. Rare extended families featuring individuals with HLHS and related CHD have been reported [10]. These families, where the reasonable supposition is that there are shared causes, emphasize that HLHS is part of a spectrum of left-sided lesions including coarctation of the aorta and congenital aortic valve stenosis [10]. Echocardiography in families shows excess appearance of subclinical cardiac anatomic defects such as bicuspid aortic valve and LV thickening in 10–20% of first-degree relatives [7,11]. These observations, which can be viewed as incomplete or variable expression of susceptibility, suggest that more subtle consequences of genetic variants in families are common.

### 1.3. Heritability

Observations of families and relatives led to publications that estimated broad sense heritability of left-sided lesions and HLHS [12,13]. Using different assumptions and independent data sets, the estimated heritability was in the range of 50–90%. These estimates are easily misinterpreted, and caution is warranted. Ascertainment biases can increase or decrease the apparent heritability. Bias of clinic-based sampling, compared to population-wide sampling, probably increases the heritability estimate due to inclusion of more severe cases and the tendency to recruit familial cases. On the other hand, meta-analysis of CHD in miscarriages and stillbirths suggests that CHD is much more common in early pregnancies compared to the live-birth prevalence rates [14]. The consequent decrease in observed CHD recurrence would lead to negative bias in heritability estimates. *De novo* mutation, an important mechanism in the genetics of HLHS, does not contribute to heritability. 

### 1.4. Epidemiology

Examining trends in fully ascertained populations consistently shows higher rates of HLHS in males compared to females and in people of European ancestry compared to other populations [15,16]. There may also be unexplained seasonality in the birth prevalence of HLHS [17,18]. These are not features expected to occur in rare monogenic disorders or those dominated by de novo mutation. Based on both standard population genetics theory and extensive empirical confirmation of that theory in other diseases over the last decade, the epidemiology of HLHS is strongly suggestive of complex and multifactorial inheritance. 

## 2. Monogenic and Chromosomal Inheritance

### 2.1. Chromosomal

Turner syndrome (45, X; monosomy X) is the most common known genetic cause of left-sided heart defects and HLHS [19], and accounts for about 2% of HLHS cases [20]. In this clear-cut example, there is evidence of complex mechanisms. Only 30% of Turner patients have CHD of any type, demonstrating that even strong genetic effects may not be determinative. There have been some efforts to map loci on the short arm of the X chromosome that influence risk of CHD [21], but progress is difficult due to extreme rarity of patients with partial deletions of Xp that could be used for fine mapping. HLHS is also seen in other chromosomal disorders, demonstrating that the cardiac phenotype can evolve from different embryological mechanisms.

### 2.2. De Novo Mutation

A birth defect like HLHS would have been near reproductive lethal in the era before surgical management. Based on classical mutation selection balance theory, it is natural to look for de novo genetic mechanisms as explanation for a sustained rate of occurrence. Copy number variants have higher mutation rates than other allele types and so it is a good research strategy to evaluate de novo CNV as possible causes of HLHS. It was known even before the era of routine microarray that deletions of 11q are associated with left sided lesions and HLHS (Jacobsen syndrome) [22]. Other studies have emphasized the excess occurrence of rare large CNV is HLHS patients [23,24,25,26]. These studies have another role in that they sometimes provide the first genetic evidence for the effect of a specific gene in the CNV interval. This is the case with Jacobsen syndrome in which there is evidence that the cardiac phenotype is primarily influenced by *ETS1.* Another recent example in the HLHS literature is a novel syndrome caused by 6q25 deletions involving *TAB2* as the likely driver gene for CHD [27,28,29].

### 2.3. Single Nucleotide Variants and Other Variant Types

Exome sequencing studies provide a potentially unbiased strategy for identifying de novo coding sequence variants underlying HLHS. There are two clinical classes of patients—those with syndrome presentations and those with isolated CHD. Kabuki syndrome (caused by de novo pathogenic variants in *KMT2D*) is an example of the former and appears to be more common that previously appreciated [30]. There are many other examples, such as Mowat–Wilson syndrome (*ZEB2*) [31], and these demonstrate that HLHS may be only one of many different anatomic and embryological types of CHD arising from specific genetic mechanisms. A listing of syndromes and genes associated with HLHS obtained by abstraction of several resources is provided in a supplemental file. The occurrence of patients with *KMT2D* pathogenic variants among apparently non-syndromic cases emphasizes both the difficulty of the clinical diagnosis and that clinical phenotypes are often incomplete without information from genetic testing [32,33,34]. Large exome studies have identified candidate de novo variants in patients with apparently non-syndromic HLHS and related heart defects [32,33,35,36,37]. The rarity and high diversity of these de novo variants serves to emphasize the potentially large number of loci that could be contributing to HLHS. 

### 2.4. Autosomal Dominant

Linkage mapping, candidate genes, and positional cloning have also contributed to understanding of rare familial forms of HLHS and left-sided lesions. *NOTCH1* has been confirmed in multiple studies to underlie some familial recurrences in which there is autosomal dominant transmission [38,39,40]. The occasional occurrence of severe left-sided lesions in known dominant disorders such as Holt-Oram (*TBX5*) demonstrates the clinical difficulty and strongly argues for unbiased sequencing strategies rather than narrow phenotype-specific panels in both research and diagnostic testing.

### 2.5. Mosaicism

In principle, parental germline mosaicism could be an explanation for sibling recurrence of severe birth defects like HLHS. In addition, embryonic and somatic mosaicism might allow an otherwise lethal variant to cause a severe developmental defect in a live-born infant. A recent case report highlights the joint use of methylation profiling and sequencing to establish mosaicism for *KMT2D* in a single patient [41]. Limitations of technology currently reduce the sensitivity of both research and clinical testing for mosaicism. We can anticipate that deeper sequencing may address this possibility more definitively in the future.

### 2.6. Locus Heterogeneity

For some diseases, a long list of different genes, each acting independently, can be found as more patients are sequenced. There are many examples where subtle informative phenotypic differences among patients were only fully appreciated once this heterogeneity was discovered. Demonstrations of rare non-recurrent CNVs, multiple singleton candidate loci with de novo variants in studies that show excess burden of de novo variants, and the lack of a clear lead locus in autosomal dominant left-sided lesions all point to very complex origins of HLHS. The failure to find one or a few monogenic disorders even considering exome or genome [42] sequencing in hundreds of HLHS patients should be viewed cautiously. The present findings do indicate that much larger case series will be required to assemble the required validation evidence and fully catalog all the possible monogenic causes of HLHS. Extreme locus heterogeneity also raises red flags that the genetic contributions to HLHS may not be exclusively high penetrance and deterministic. Alternative inheritance mechanisms require different methods of analysis and types of evidence.

## 3. Complex Inheritance

### 3.1. Transmitted Deleterious DNA Variants

A consistent observation in genetic studies of HLHS and congenital heart defects, in general, is the observation of what appear to be functionally deleterious variants in genes located in known cardiac developmental pathways but transmitted from unaffected parents [12,32,33,37,42]. Even with convincing functional evidence, proof that one of these variants plays a causal role in an individual case is difficult. The possibility of oligogenic inheritance is particularly challenging. In an oligogenic mechanism, deleterious alleles in two or more genes act together to cause a heart defect while none of them by themselves would be sufficient. Statistical methods for establishing oligogenic inheritance are being developed in parallel with other methods for rare variant association testing [43,44] and suggestive cases have been described [45,46].

### 3.2. Common Variants

**Figure 1:**
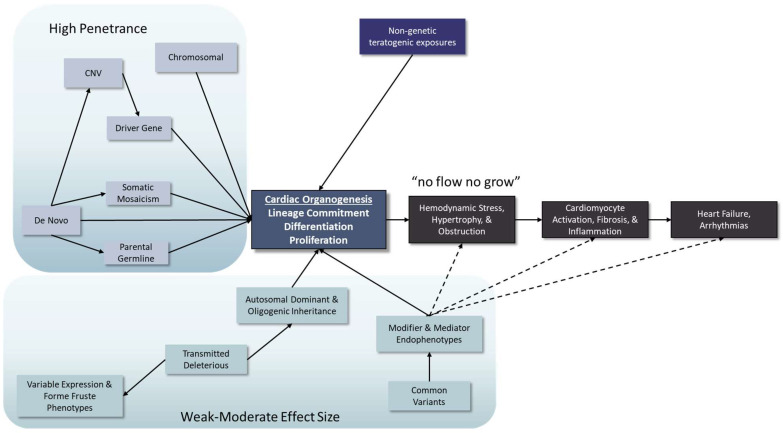
The known genetic causes of HLHS are complex. They include highly penetrant effects of pathogenic DNA variants where the presence of the genetic abnormality is determinative for the heart defect. There is also substantial evidence for weaker genetic influences in which both rare and common DNA variants contribute to increase the risk of the disorder. Cell differentiation, cell functional defects, and their associated anatomic abnormalities set the stage additional short- and long-term complications, which may also be influenced by genetic effects.

There is also evidence, albeit incomplete, that common genetic variants contribute to left sided congenital heart defects [47,48,49,50,51]. Genome wide association studies have found loci that meet standard statistical criteria for significance, but the loci identified have not yet overlapped or been strongly validated. New well-powered studies utilizing the UK Biobank data have shown convincing genetic effects on cardiac valve and aortic endophenotypes that can be directly related to left heart and outflow tract development [52,53]. In a striking recent example of this research strategy, Tcheandjieu et al. [53] used MRI imaging data from the UK Biobank to estimate ascending aortic diameters, mapped common gene variant associations, and used those results to make a polygenic score. They found a decrease by one standard deviation in aortic diameter score was associated with an increased risk of congenital left sided heart defects but not with other subtypes of CHD. These studies suggest not only important common variant effects on the risk of left-sided CHD but also risk of some of the most clinically important complications (see Figure 1). 

### 3.3. Non-Genetic Causes

**Figure 2:**
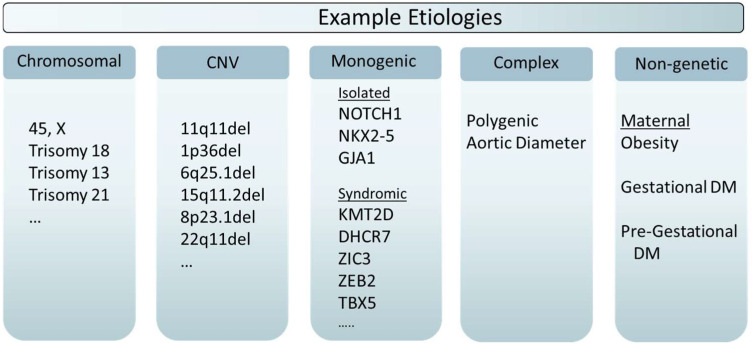
Examples of specific HLHS-associated genetic disorders or risk factors. These lists are not comprehensive and meant only to illustrate some of the more commonly encountered disorders. The syndrome associations listed include: *KMT2D*—Kabuki syndrome; *DHCR7*—Smith-Lemli-Opitz syndrome; *ZIC3*—X-linked Heterotaxy; *ZEB2*—Mowat–Wilson syndrome; *TBX5*—Holt-Oram syndrome.

Non-genetic factors may impact the same cells and developmental processes as genetic variants [18]. Identification of environmental agents that cause birth defects can start with cell and model organism studies but ultimately requires observational epidemiology to establish associations in the general population. A grouping of maternal risk factors that includes obesity [54], gestational diabetes mellitus [55,56], and pre-gestational diabetes [56,57] have shown positive associations with the occurrence of HLHS and related left-heart malformations. These same risk factors impact risk for other forms of CHD, particularly heterotaxia. This suggests that maternal metabolic derangements can affect embryonic cardiac organogenesis, but the precise mechanisms are not understood. Because of the many methodological challenges, replication is essential. A recent umbrella analysis of meta-analyses [58] has provided strong support for the consistency of these risk factors and other exposures. Continued active surveillance of HLHS in the population is warranted. Figure 2 gives an overview with examples of some the accepted causes of HLHS.

## 4. Clinical Implications of Genetic Testing

### 4.1. Clinical Utility

There is substantial evidence of clinical utility for comprehensive genetic testing in the literature and that has led to expert recommendations, professional society guidelines, and adoption by a growing number of payers [59,60,61]. The domains of utility for genetic testing are similar to clinical imaging. Potential utilities include precision diagnostic thinking, improved prognostic certainty, tailored management plans, selection of targeted therapeutic strategy, personal utilities in counseling and family cascade testing, and improved health system planning. There is a general recognition that the final standard for evidence-based medicine should be improved patient outcomes including survival and quality of life. Because those studies take many years to perform, there is not yet definitive evidence for positive effects of precision diagnosis on patient outcomes. There is not yet evidence for improved patient outcomes in congenital heart disease or hypoplastic left heart syndrome, in particular. However, there are promising avenues for future exploration. Cell therapies, prenatal valvuloplasty, pharmacological therapies, and hyperoxygenation are potential treatments for HLHS [62]. Heterogeneity of treatment effects may be partially explained by whether the child has an identifiable genetic disorder.

### 4.2. Possible Harms and Disutility

As with any novel medical technology, one may reasonably ask whether there are predictable or frequent harms from genetic testing. This topic that has been addressed primarily from the point of view of ethics and leading to generally accepted professional guidelines [63,64]. Clinical risks of genetic testing are mainly in misdiagnosis (attributing the clinical phenotype to an incorrect disease) and overdiagnosis (inferring that features of a disease will occur in the future but that never materialize). The situation has markedly improved with the near universal adoption of ACMG/AMP guidelines for assessing pathogenicity of DNA variants, as well as interpretation and reporting standards [65]. These challenges are not unique to genetic testing and careful physicians should use the results as part of an integrated approach to diagnosis and clinical decision making. Genetic test results are an aid to diagnosis and cannot stand alone outside the clinical context of the case and family.

### 4.3. Cost Effectiveness

An important policy question is whether and under what circumstances hospitals and health systems should view comprehensive genetic testing as medical necessary. The main factors that one might consider are (1) superior technical performance compared to standard care; and (2) cost-effectiveness. There is already substantial support for the superiority of exome and genome sequencing compared to standard precursor genetic testing. At a purely technical and lab interpretation level exome and genome can detect essential all DNA variant types that are typically reported clinically and thus outperform all previous single tests [66,67]. Exome and genome sequencing also solve a major clinical problem in that even experienced physicians cannot reliably arrive at specific diagnoses of rare and ultra-rare disorders without confirmation by lab testing. This has been demonstrated over and over in the literature in which patients with non-specific but severe clinical presentations achieve precision diagnoses with exome or genome testing. 

Genomic data management is getting easier and less costly with the advent of convenient cloud solutions. Storage of raw genomic information in the EHR or any other local server is neither necessary nor desirable. The rapid advance of data quality coupled with dramatic cost reductions will continue to smooth the transition from limited targeted sequencing to unbiased genome technical platforms. Interpretation of exomes and genomes is also improving and, fundamentally, the challenge of variants of unknown significance is no different than that encountered in large panels. The development of fast and accurate AI methods for variant prioritization is already reducing the hours required for professional level analysts to review long lists of DNA variants. Unlike in research aimed at novel gene discovery, clinical reporting is necessarily conservative and focused.

There is a growing literature on the cost effectiveness of exome and genome sequencing [68,69,70,71]. The main focus of these publications is on measures of cost-per-diagnosis and the literature does not yet address total effects on cost-of-care. Early studies point to the possibility of reduced lengths of stay in hospital but need confirmation [59]. All this literature supports the cost-effectiveness of diagnostic exome and genome sequencing and in some clinical contexts suggest cost-savings. An analysis of cost effectiveness has not yet been performed focusing on infants with congenital heart disease. It is likely to be much more useful if offered in early infancy where precision diagnosis can have the biggest impact on clinical decision making.

### 4.4. Clinical Data Sharing

Because HLHS is a rare disorder and many of the underlying causes are ultra-rare, aggregation of genetic data should be an important future goal. Currently, the best such resource is ClinVar [72] which asks that labs voluntarily contribute variants that have been classified as to their apparent pathogenicity across a wide spectrum of genetic disorders. A deficiency of ClinVar is that it does not collect complete and structured case-specific phenotypes. The lack of detailed phenotypes inhibits integrative research and much more needs to be done in this arena. An effort to share clinical genetic data across institutions could produce substantial returns by solidifying the evidence around very rare disorders.

## 5. Next Steps in Research

### 5.1. Large Scale WGS

Future studies must incorporate the probable independent and joint roles for rare and common genetic variation. As costs of individual genome sequencing have plummeted, it will be increasingly attractive to use that method since it provides the coding sequence coverage of exomes, but also provides all of the non-coding variation. Short read genome sequencing is already a platform from which to identify almost all the known DNA variant types including some structural variants and repeat expansions. Very large studies, perhaps requiring tens of thousands of patients, and utilizing genome sequencing in case parent trios will likely be needed to fully catalog single gene effects and their modifiers.

### 5.2. Phenotypic Heterogeneity and Integrative Studies

HLHS has several distinct clinical presentations (slit-like, thickened and obstructive remnant left ventricle, and miniature [73]) that have not been fully characterized for their genetic correlates. New integrative technologies show great promise to refine the molecular phenotypes of congenital heart defects at the level of cardiac and vascular tissue. These methods can show not only the signatures of the original causal effects but also provide biomarkers of progression and severity. A recent striking example is provided by Hill et al. [74] where single nucleus sequencing from hard to acquire cardiac tissues showed an abnormal population of activated cardiac fibroblasts in HLHS patients. Future studies that combine molecular phenotyping, such as methylation, ATACseq, and single nucleus RNAseq profiling should be integrated with genome sequencing. Such precision phenotyping may relieve some of the requirements for huge sample sizes that come with complex genetics.

### 5.3. Exploratory Long Read WGS

Genome sequencing technology is also moving very quickly to include long read sequencing (10–100 s of kB). These methods can give more accurate and higher sensitivity detection of large structural variants that are often invisible to short read sequencing and where the accuracy is very low [75,76]. It is unknown how many patients are affected by these variants, but it is important to have a more definitive catalog from well-defined patient populations and to evaluate whether there are any prominent recurrent defects that have so far escaped detection.

### 5.4. Clinical Utility of Newborn WGS

Finally, it is important that all the recent discoveries about the genetic causes of HLHS are brought into routine clinical practice. It is especially important to detect syndrome diagnoses early as they can be very difficult to recognize and may have a large effect on neurodevelopmental outcomes. Recent publications have demonstrated the efficacy of genome sequencing in newborns and have also shown evidence for clinical utility [58,59,76]. Genome sequencing replaces batteries of serial genetic tests and gives a single test option to evaluate for all the mechanisms discussed here. Pediatric cardiologists should become familiar with ordering comprehensive clinical genetic tests, such as exome and genome sequencing, when necessary for patient benefit.

## Data Availability

Not applicable.

## Supplementary Materials

The following supporting information can be downloaded at: https://www.mdpi.com/article/10.3390/jcdd9100315/s1. References [77,78,79] are cited in the supplementary materials.
